# Fructose Metabolism and Relation to Atherosclerosis, Type 2 Diabetes, and Obesity

**DOI:** 10.1155/2015/823081

**Published:** 2015-06-14

**Authors:** Astrid Kolderup, Birger Svihus

**Affiliations:** ^1^Faculty of Public Health, Hedmark University College, P.O. Box 400, 2418 Elverum, Norway; ^2^Norwegian University of Life Sciences, P.O. Box 5003, 1432 Aas, Norway

## Abstract

A high intake of sugars has been linked to diet-induced health problems. The fructose content in sugars consumed may also affect health, although the extent to which fructose has a particularly significant negative impact on health remains controversial. The aim of this narrative review is to describe the body's fructose management and to discuss the role of fructose as a risk factor for atherosclerosis, type 2 diabetes, and obesity. Despite some positive effects of fructose, such as high relative sweetness, high thermogenic effect, and low glycaemic index, a high intake of fructose, particularly when combined with glucose, can, to a larger extent than a similar glucose intake, lead to metabolic changes in the liver. Increased *de novo* lipogenesis (DNL), and thus altered blood lipid profile, seems to be the most prominent change. More studies with realistic consumption levels of fructose are needed, but current literature does not indicate that a normal consumption of fructose (approximately 50–60 g/day) increases the risk of atherosclerosis, type 2 diabetes, or obesity more than consumption of other sugars. However, a high intake of fructose, particularly if combined with a high energy intake in the form of glucose/starch, may have negative health effects via DNL.

## 1. Introduction

Sugars are important sources of energy in our diet, and a high intake of sugars has increasingly been identified as a considerable cause of major diet-induced health problems, namely, atherosclerosis, type 2 diabetes, and obesity [1–4]. An increased intake of sugar sweetened beverages in particular has been associated with these health problems in many epidemiological studies [5–7]. The main constituents of sugars are fructose and glucose, which can be present either alone or in combination, although most commonly fructose is mixed 50 : 50 with glucose. The ratio of glucose : fructose in the diet has been argued to be important for some of the effects of sugars [8, 9]. Thus, it is important to study the effects of fructose and glucose together, but also it is important to study the effects of fructose alone. Fructose has been claimed to be of benefit because it may aid glycemic control [10, 11], but also it has been claimed to be more harmful than other sugars, especially with regard to the development of atherosclerosis, type 2 diabetes, and obesity [8].

Fructose is found in a variety of foods. In table sugar, it is bound to glucose to form the disaccharide sucrose, whereas in honey it occurs in monosaccharide form. In fruit, berries, and vegetables, fructose occurs in both monosaccharide and disaccharide forms. Measured as intake from caloric sweeteners in USA, fructose intake was rather stable throughout the fifties and sixties but increased from the seventies until the end of the nineties, after which intake has declined [12]. The average American fructose intake was estimated to be 49 g per day in 2004 [13]. In Norway, the average daily intake of fructose can be estimated to be approximately 56 g/day, based on data from the Norwegian Directorate of Health's survey of consumption patterns [14] and composition data [15]. Globally, the main source of fructose is sucrose, which constitutes >90% of the energizing sweeteners used in the world [16]. In some countries, such as USA and Japan, high-fructose corn syrup (HFCS) is also an important source of fructose. HFCS is a mixture of fructose and glucose in different concentrations. It can contain up to 90% fructose [17], but the dominating concentration of fructose is 42% or 55% in commercial products [9]. In USA, consumption of HFCS increased sharply from 1970 until 1999, but it has declined since then [12, 18].

This review paper aims to describe how fructose, compared with other sugars, is managed by the body, thus clarifying the impact of fructose on atherosclerosis, type 2 diabetes, and obesity.

## 2. Materials and Methods

The review is based on scientific peer-reviewed papers obtained using a nonsystematic search of the databases PubMed and Web of Science. The first step in the selection of literature was to identify the relevant keywords to search for in these databases. Various combinations of the following keywords were used: “fructose,” “glucose,” “sucrose,” and “sugar” combined with “metabolism,” “insulin resistance,” “overweight,” “obesity,” “relative sweetness,” “absorption capacity,” “glycemic index,” “de novo lipogenesis,” “thermogenesis,” “type 2 diabetes,” and “appetite regulation.”

The next step in the selection process consisted of inclusion or exclusion of papers based on the relevance to the study's aim. Through this initial selection process, both original and review papers were included. The reference lists of the papers were thoroughly studied to discover any possibly relevant papers that were not included. As far as possible, only original papers and reviews based on controlled trials and mechanistic studies were included. Unless otherwise specified, the results presented and discussed in this paper are statistically significant, and the experiments were conducted with a control group. Although animals may metabolize fructose differently from humans, animal studies have been included when found relevant, for example, due to lack of human studies. One possible limitation in this study lies in the nonsystematic nature of the search strategy, resulting in the possibility that relevant papers were not considered.

## 3. The Body's Fructose Management

### 3.1. Absorption

Although there is some uncertainty about the mechanisms of fructose absorption, most of fructose seems to be absorbed by facilitated transport in the jejunum by the fructose transporter GLUT5 [18–20]. The body has limited capacity to absorb pure fructose, and intake of fructose can therefore lead to malabsorption [20–24]. Malabsorption of fructose results in bacterial fermentation, which leads to formation of short-chain fatty acids (acetate, propionate, and butyrate) and gases (hydrogen, methane, and carbon dioxide) [25, 26]. These processes can affect the motility of the intestine and cause various symptoms such as abdominal pain, bloating, and altered stool [27]. A large individual variation in absorption capacity for fructose has been observed. When fructose is consumed as a single oral dose, the maximum absorption capacity has been shown to vary between 5 and 50 g [28]. Several factors seem to affect this capacity, such as age and health [27, 29, 30], but of the dietary factors the presence of glucose is the most important [20, 23]. A significant increase in fructose absorption has been shown when fructose is coingested with equal amounts of glucose [21, 31]. There is still uncertainty about how glucose increases absorption capacity for fructose, but it may amongst others be due to an effect of glucose on the presence of fructose transporters [20]. Fructose does not appear in any fruit or vegetable without glucose [15]. Evolutionarily, this may explain why humans do not have the ability to absorb large amounts of pure fructose.

### 3.2. Metabolism

After absorption, fructose is transported by the portal vein to the liver, where it is effectively absorbed by liver cells [32], resulting in only small amounts entering the systemic circulation. The concentration of fructose in the blood is therefore only about 0.01 mmol/L, unlike that of glucose which is approximately 5.5 mmol/L [33]. Metabolism of fructose thus occurs primarily in the liver, but fructose may also be metabolized by enterocytes. In a study of pigs, it was shown that intestinal lactate production from fructose could account for 12% of absorbed fructose [34], but the functional significance of intestinal metabolism of fructose in humans remains unknown [18]. Bjorkman and Felig [35] also showed that infused fructose (48.6 g fructose in liquid form infused intravenously) was metabolized in the kidneys in humans who had fasted for 60 hours. Despite the artificially high blood fructose level, that study still shows that the kidneys have a relatively large capacity to metabolize fructose. It has been shown that GLUT5 is expressed in the membrane of fat, kidney, muscle, and brain cells [32, 36], but, due to very low levels of fructose in the blood, negligible amounts of fructose are probably metabolized in these tissues [9, 18].

As discussed above, the liver will metabolize a large majority of the ingested fructose, compared to only about 15–30% of ingested glucose [37, 38]. Most of the reactions in liver fructolysis are the same as those occurring in glycolysis, but fructose enters at a later stage in the glycolytic reaction chain than glucose [39]. Thus, fructose avoids the main control step in glycolysis, the phosphofructokinase step, which is tightly regulated by the energy status of the cell. The first step in fructolysis is the phosphorylation of fructose by fructokinase to fructose 1-phosphate. Unlike the phosphofructokinase in glycolysis, this enzyme is not inhibited by ATP [40]. The enzyme is considered virtually unregulated and, even when the liver's energy status (ATP) is high, the fructokinase will metabolize fructose to fructose 1-phosphate. Furthermore, fructose 1-phosphate is cleaved to dihydroxyacetone phosphate and glyceraldehyde by the enzyme aldolase B. Glyceraldehyde may be phosphorylated by triose kinase, with ATP as the phosphate donor, to form the glycolytic intermediate glyceraldehyde 3-phosphate [41]. After these steps, the carbon atoms from fructose follow the glycolytic steps.

Bypassing the phosphofructokinase step makes the flow of fructose carbon atoms through the biochemical pathways less controlled than for glucose. In this way, the liver will metabolize fructose in an unlimited way, as opposed to the case of glucose. This will influence the type and amount of metabolic products produced by the liver and is the main reason why fructose and glucose have different metabolic effects.

In the liver, fructose can enter metabolic pathways: it can be oxidized, converted to glucose (and glycogen), or converted to lactic acid, or enter* de novo* lipogenesis (DNL). After an overnight fast, approximately 50% of the fructose eaten as an oral dose of approximately 30–70 g is converted to glucose via gluconeogenesis [42]. Others have shown that after a similar fructose intake about 45% is oxidized; however, this includes both direct fructose oxidation and oxidation of glucose and lactic acid formed from fructose [43]. Another metabolic fate of fructose is to form lactic acid, but this seems to occur only at high fructose intakes [44–48]. The ingestion of 72 g fructose is the lowest amount that has been experimentally demonstrated to result in lactic acid formation [49]. Intake of fructose may also lead to formation of fatty acids via DNL. The diet composition may influence the distribution of fructose. Theytaz et al. [50] showed that coingestion of glucose with fructose decreased oxidation and gluconeogenesis from fructose carbon atoms. Intake of fructose together with glucose thus seems to affect the metabolic fate of fructose. To some degree, this effect may be due to higher insulin secretion after intake of glucose compared to fructose [51]. Insulin will, amongst others, decrease glucose production from fructose [52], and insulin will also stimulate DNL [53]. The extent to which fructose enters DNL is central to the health effects of fructose.

### 3.3. *De Novo* Lipogenesis (DNL)

DNL is the metabolic pathway that transforms surplus nonfat energy into fat by synthesis of fatty acids from acetyl-CoA [54]. The liver is the primary site of DNL, but DNL can also occur in lactating mammary glands and adipose tissue. Key lipogenic enzymes are present in adipose tissue, but to what degree adipose tissue contributes to the total DNL seems to be unclear. Although some data indicate that DNL in adipose tissue is of little importance [55–57], it has also been shown that it may play a more significant role [58], especially after ingestion of large amounts of carbohydrates [59, 60]. Some controversy exists regarding the capacity of the liver to carry out* de novo* lipogenesis in humans [60, 61], although a high capacity has been demonstrated in several experiments [62, 63].

As a result of the metabolic difference between glucose and fructose, a higher percentage of fructose compared to glucose can be converted to fat in the liver via DNL [63]. This has been shown in a number of animal and human studies, in which these sugars have been consumed in equal quantities under similar experimental conditions [63–66]. The greater potential of fructose to stimulate DNL is the main reason why fructose has been portrayed as particularly harmful. As described earlier, the liver absorbs most of the ingested fructose, and fructose metabolism bypasses the main control step in glycolysis, which means that a greater proportion of fructose, compared with glucose, is available for DNL. A normal diet, however, provides 3 to 5 times more glucose than fructose [12, 67], and this may influence the practical relevance of this metabolic difference. If, for example, glucose intake is very high, the liver may need to handle larger amounts of glucose than fructose. At two points in liver fructolysis, intermediates can enter lipid synthesis [9]. Fructose is converted to dihydroxyacetone phosphate, an intermediate in equilibrium with glycerol 3-phosphate, which forms the basis for the glycerol in triglycerides and phospholipids. Meanwhile, a large proportion of fructose carbons are metabolized directly to pyruvic acid and then acetyl-CoA. On metabolism of a large amount of fructose to acetyl-CoA, the amount of acetyl-CoA may exceed the citric acid cycle capacity of mitochondria. High levels of fructose may thus act as a nonregulated source of hepatic acetyl-CoA, which is a substrate that can enter DNL [9]. In addition, a high intake of fructose seems to stimulate gene expression and activity of lipogenic enzymes in the liver [40, 41, 66, 68]. Recently, fructose has also been shown to give a higher increase in fibroblast growth factor 21 (FGF21) than glucose. FGF21 is a hormone involved in glucose and lipid homeostasis [69], and high levels of FGF21 seem to be associated with metabolic disease [70].

It is clear that a high intake of fructose will cause a significant increase in DNL activity [71]. However, there is a paucity of knowledge about the effect of a normal fructose intake on this activity [72]. No research seems to have been conducted to assess the minimum amount or individual range of fructose that must be eaten to obtain a significant increase in DNL, which among others may be due to difficulties in quantifying DNL. In fact, measurement of hepatic DNL must be considered semiquantitative [54, 73]. Chong et al. [73] found that ^13^C-labelled fructose contributed only 0.4% of the triglycerides in very-low-density lipoproteins (VLDLs) in men. This was measured 6 hours after eating a meal of 0.5 g fat/kg body weight and 0.75 g fructose/kg body weight after an overnight fast. The small increase in triglyceride levels in the blood after a fructose intake may be a result of delayed triglyceride secretion. Incorporation of carbon from ^13^C-labelled fructose in VLDLs will not always reflect DNL, because some of the newly synthesized fatty acids will have delayed secretion [74, 75]. The level of DNL activity after fructose intake seems to vary significantly among individuals [76–78], and it also seems to vary during the day [79, 80].

Excessive intake of fructose, and hence increased DNL, may increase the risk of disease, because it may potentially cause both increased cholesterol levels in the blood and accumulation of fat in the liver [81]. In several animal studies, fat accumulation in the liver has been demonstrated after a high intake of fructose [82, 83], although most human studies have failed to demonstrate such an effect [84–86]. In fact, Chiu et al. [87] concluded, based on a systematic review with meta-analysis of controlled feeding trials, that fructose does not increase lipid content in liver when isocalorically exchanged for other carbohydrates. However, they pointed out that fructose ingested in large doses can raise liver lipid content, an effect that may be due to excess energy rather than fructose per se. Bravo et al. [88] conducted a study where participants consumed sucrose or HFCS for ten weeks at different levels of intake. In the study, fructose consumed as a part of a normal diet did not promote increased liver lipid content even at high intakes (90th percentile consumption level). In a study referred to by Rippe and Angelopoulos [89], no increase in liver fat was observed after consumption of HFCS or sucrose at levels up to 30% of energy for ten weeks. However, Maersk et al. [90] found that intake of 1 L sucrose-sweetened soft drink per day for six months increased liver lipids in overweight participants compared with intake of same amount of milk, diet cola, and water. This may illustrate that the combination of fructose and glucose, as in sucrose, can lead to increased level of liver lipids. However, it is not possible to conclude that this is an effect of fructose, since there was no glucose control group in this study. Johnston et al. [91] found no difference in liver triglyceride level between overweight participants that ate a hypercaloric high-fructose or high-glucose diet. The authors concluded that this result indicates that the hypercaloric state rather than macronutrient composition is important for the accumulation of liver lipids. Carbohydrate-induced accumulation of fat in the liver can lead to nonalcoholic fatty liver disease (NAFLD) [92]. However, it is still unclear if fructose via increased DNL is particularly conducive to NAFLD [93]. The effect of fructose on lipid accumulation is thus unclear, but the effect of fructose on the blood lipid profile seems to be better documented.

## 4. Atherosclerosis

It appears that high fructose intake can create an unfavorable lipid profile in blood via DNL [94]. The main product of DNL is palmitic acid [95], a fatty acid specifically shown to increase the risk of atherosclerosis [96]. Fatty acids formed by DNL will mainly be packed in VLDLs delivered into the bloodstream. This may, in turn, increase the level of low-density lipoproteins (LDLs) in the blood. In several studies, fructose has to a greater extent than glucose increased blood levels of triglycerides [51, 65, 97, 98] and LDLs [65, 99–103]. Aeberli et al. [104] showed that fructose increased the small dense LDLs, the type of LDLs that may in particular be linked to cardiovascular risk [105]. The level of high-density lipoproteins (HDLs) in blood does not seem to be affected by fructose [100, 101]. In most studies, an intake of fructose >100 g/day has been necessary to observe the adverse effects on lipid profiles [51, 65, 85, 98, 100, 106–108]. However, in a recent study by Aeberli et al. [109], a daily intake of about 77 g fructose and 34 g glucose for 3 weeks resulted in increased levels of total cholesterol and LDLs in the blood of healthy young men, compared with a daily intake of approximately 109 g glucose and 28 g fructose over the same time period. The fact that both groups also ingested an unknown amount of starch and the fact that food intake was not controlled reduce the solidity of these results. Maersk et al. [90] found that intake of 50 g fructose per day together with 50 g glucose could have a negative effect on blood triglyceride level. The lack of a control group ingesting glucose makes it difficult to conclude that this is an effect of fructose. Conversely, Lowndes et al. [110] found no negative effect on lipid profile in overweight or obese individuals consuming HFCS or sucrose incorporated in a eucaloric diet for ten weeks at levels corresponding to the 25th and 50th percentiles of adult fructose consumption. Using the current knowledge, it does not appear that the consumption of moderate amounts of fructose (<50 g/day) alone will result in an unfavorable blood lipid profile [86, 111].

Due to the insignificant levels of fructose in peripheral blood, as described above, only glucose has the potential to be a substrate for DNL in adipose tissue. Although DNL in adipose tissue seems to be small as earlier discussed, glucose will, because of its presence in blood and by raising blood insulin level, probably to a higher extent than fructose, stimulate DNL in adipose tissue. Intake of glucose, in amounts that exceed the total capacity for glycogen storage and glucose oxidation, may thus increase DNL in adipose tissue more than the same amount of fructose. While fat formed in the liver has to be transported as lipoproteins in blood, this is avoided if the fat is formed directly in adipose tissue. Considering known negative health effects of lipoprotein residues, DNL occurring in adipose tissue may be preferable compared with DNL in the liver. This may illustrate a metabolic difference between glucose and fructose when consuming large amounts of sugars.

Another possible difference between fructose and glucose on risk factors for atherosclerosis is the effect of these sugars on blood uric acid level. Increased uric acid level has been associated with atherosclerosis in epidemiological studies, but the causality is uncertain [112–114]. Fructose appears to increase uric acid levels in the blood to a higher extent than glucose, especially at high intakes and when consumed as excess energy [86, 115, 116]. Intake of 0.5 g fructose/kg body weight is the lowest quantity shown to result in uric acid formation [117]. An increased blood level of uric acid can theoretically lead to elevated blood pressure because uric acid inhibits an enzyme in the endothelial cells of the arteries called endothelial nitric oxide synthase (eNOS). Activated eNOS leads to increased production of nitric oxide (NO), an important vasodilator. Thus, inhibition of eNOS may lead to vasoconstriction. Although only 0.5 g fructose/kg body weight has been shown to give uric acid formation and increased uric acid level theoretically could increase blood pressure, results from studies of the effect of fructose on blood pressure are very inconsistent [65, 72, 118, 119]. An average intake of fructose does not seem to lead to increased blood pressure [111, 120, 121]. The lack of causal link between uric acid level and atherosclerosis makes it difficult to draw conclusions on this effect of fructose.

## 5. Type 2 Diabetes

A high intake of sugar-sweetened beverages, with fructose as one of the major types of monosaccharides, has been associated with development of type 2 diabetes [5, 122]. Although this association does not prove causation, it is important to study the role of fructose in the development of type 2 diabetes. Central to the understanding of type 2 diabetes is the effect of nutrients on blood glucose homeostasis. Fructose must be converted to glucose in the liver to cause an increase in blood glucose level. As the conversion takes time and only a portion of the fructose will form glucose, fructose increases blood glucose less than similar levels of glucose [51]. Thus, the glycemic index for fructose is only 23 [10]. This, together with lack of stimulation of the pancreatic *β* cells [123], gives lower insulin secretion after intake of fructose compared with glucose [51, 124, 125]. These effects are positive because they contribute to blood glucose homeostasis. Additionally, moderate amounts of fructose have been shown to have positive effects on glycemic control [86, 126, 127]. However, it is claimed that fructose may also contribute negatively to blood glucose homeostasis by causing insulin resistance in the liver [9]. There is evidence that a high intake of fructose can cause insulin resistance in animals [128, 129], but several human studies have failed to demonstrate such an association [103, 130–132]. The operational definition of insulin resistance or sensitivity seems unclear, and many different methods have been used to measure it [133]. Thus, it is difficult to compare studies of insulin resistance. In human studies, in which fructose has been reported to cause insulin resistance, the daily intake of fructose has been as high as 110 g [109], approximately 250 g [134], 80 g [135], and 138 g [65]. This may indicate that the fructose intake must be high to potentially cause insulin resistance [86]. In the studies by Aeberli et al. [109], Stanhope et al. [65], and Beck-Nielsen et al. [134], total food intake was not controlled. Thus, the observed effect of fructose may also have been caused by differences in food intake between the control and experimental groups. In all the studies, in which insulin resistance has been shown, fructose was eaten together with glucose or starch, so the observations could also be the result of a combination of fructose and glucose. A number of hypotheses on how fructose can cause insulin resistance in the liver have been proposed. Lipid accumulation in the liver [136–138], metainflammation [83], and oxidative stress [139] are, either via inhibitory phosphorylation of the insulin receptor or the signaling molecules involved in insulin signaling, possible mechanisms for fructose-induced insulin resistance [9]. However, there are too few studies in humans and these are too divergent to be able to conclude firmly that there is a link between the consumption of fructose and insulin resistance. More long-term studies in which the daily intake of fructose is moderate are needed.

## 6. Obesity

It is debatable whether fructose is less satiating than other sugars and thus can contribute to obesity through a high food intake. In a study by Page et al. [92], magnetic resonance (MR) images were taken of human brains after giving 75 g of a fructose or glucose drink. Glucose, but not fructose, reduced activity (regional cerebral blood flow) in the hypothalamus in the areas involved in energy regulation and reward systems; this is probably an indicator of satiety and may indicate that fructose is less satiating than glucose. Fructose will also to a lesser extent than glucose increase blood levels of insulin [51], leptin [51, 140], gastric inhibitory polypeptide [141], and glucagon-like peptide-1 [92], while at the same time it will attenuate levels of ghrelin less [51]. Although these hormonal effects may indicate that fructose is less satiating than glucose, this has not been confirmed in studies of the ability of fructose to satiate. In such studies, it has been shown that fructose has a greater appetite-reducing effect than glucose, when intake occurs before a meal [142, 143], or that there is no difference in the effects on appetite between fructose and glucose [144, 145]. Therefore, the effect of fructose on appetite remains unclear.

Although it is conceivable that fructose, via lack of stimulation of satiety signals, could contribute to obesity, fructose has several properties that act against obesity. As previously mentioned, the small intestine has a limited capacity to absorb fructose. This can lead to malabsorption at least if large amounts are consumed and consumption occurs without glucose-providing nutrients. Malabsorption of fructose will make less fructose enter the bloodstream and thus less energy will be available to the cells. In this way the malabsorption will act against obesity. It has also been shown in numerous studies that fructose has a greater thermogenic effect than glucose [46, 146–148]. This means that the body uses more energy after eating fructose rather than glucose, so less energy will be available to be stored as fat. The relative sweetness of fructose is also greater than for glucose and sucrose [149, 150]. Although this will decrease with increasing temperature [151, 152], the high relative sweetness allows smaller amounts of fructose than glucose and sucrose to be used to achieve a particular sweetness in most applications. On the basis of these properties, it does not appear that fructose is more fattening than other sugars. This also agrees with experimental studies of the relationship between fructose intake and obesity in animals [153, 154] and humans [65, 86, 106, 155].

## 7. Substrate Oxidation

It has been proposed that fructose can inhibit lipid oxidation [146]. For liver lipid oxidation this is logical, because the liver acquires energy from fructose and thus does not need to oxidize fat. Fructose can also increase DNL. It would not be expedient to form and break down fat simultaneously in the liver, and increased levels of malonyl-CoA (due to active DNL) will inhibit *β*-oxidation [41, 156]. It would also be logical for fructose to increase total body lipid oxidation more than glucose due to fructose's smaller contribution as an extrahepatic energy source. In some studies, however, fructose has been shown to increase the respiration quotient (RQ), the ratio of CO_2_ exhaled to O_2_ consumed, [157] more than glucose. This indicates that fructose to a higher degree than glucose reduces total body lipid oxidation and increases total body carbohydrate oxidation. Blaak and Saris [158] conducted a study in which participants ate 75 g fructose, starch, or glucose after a 12-hour fast in a crossover study. Fructose resulted in a significantly larger increase in the RQ, measured 6 hours after ingestion, than both glucose and starch. Tappy et al. [146] also showed a greater increase in RQ 4 hours after ingestion of 75 g fructose, compared with similar healthy participants eating 75 g glucose. Schwarz et al. [46] conducted a similar study (75 g fructose/glucose) with the same result. These results may be explained by the fact that fructose, more than glucose, enters DNL under specific conditions. Due to the fact that RQ varies for different substrates (e.g., 1 for carbohydrates and 0.7 for fats), RQ is used to determine the source of substrate oxidation [159]. However, during active DNL, CO_2_ is produced without using O_2_. Simultaneous occurrence of DNL and carbohydrate oxidation can lead to RQ values greater than 1 [159]. An increased RQ caused by DNL can, therefore, be misinterpreted as reduced lipid oxidation and increased carbohydrate oxidation. Such a misinterpretation may have occurred in the studies described above. Thus the effect of fructose on total body substrate oxidation remains unclear.

## 8. Discussion

The distribution of fructose into metabolic pathways, especially DNL, is of key importance to the health effects of fructose. The distribution varies with the amount of fructose consumed, the duration of fructose exposure, the composition of diet/meal, and whether the measurement took place postprandially, after absorption, or under fasting conditions. Individual physiological, enzymatic, and endocrine factors are also important. Diet composition and the amount of fructose eaten and absorbed will be the focus of this discussion.

Malabsorption of fructose will affect the amount of fructose absorbed and can thus be an important confounding factor in studies in which factors that affect absorption capacity have not been taken into account [39]. Truswell et al. [21] showed that intake of 50 g fructose led to malabsorption in over 50% of the study participants. The results of studies in which fructose is malabsorbed can thus be inaccurate due to individual differences in the absorption capacity of fructose. As the small intestine has a large absorption capacity for glucose and a limited one for fructose, it is problematic to compare fructose with glucose as the sole carbohydrate source. In future studies, this should be controlled for, for example, by using the hydrogen breath test to assess fructose malabsorption.

The composition of diet and especially the amount of glucose/starch may have influence on the health effects of fructose. As fructose is present with glucose in most food products, it is more practical and relevant to look at the effects of fructose and glucose together than the effects of fructose alone. A larger increase in DNL after eating fructose and glucose together (50 : 50 glucose : fructose) rather than the same amount as pure glucose has been shown [63]. Eating glucose with fructose is likely to affect fructose's health effects by stimulating the flow of fructose to DNL [50]. This effect could be due to both increased absorption capacity for fructose when coingested with glucose and therefore greater availability of fructose carbon atoms going towards DNL and increased blood insulin levels when glucose is present in the diet. Insulin stimulates DNL directly and indirectly by inhibiting other important metabolic pathways for fructose, such as gluconeogenesis. It is also plausible that glucose will compete with fructose as an energy source for enterocytes and hepatic cells, thus making more fructose available for DNL. Coingestion of glucose with fructose has been shown to decrease oxidation and gluconeogenesis from fructose carbon atoms [50]. Thus, it appears that the combination of fructose and glucose is particularly unfortunate, although fructose is not a prerequisite for DNL. It is unclear how much glucose/starch must be included with fructose for this effect to be significant.

The amount of fructose consumed seems to be of great importance to the effects of fructose on health. The negative health effects of fructose have mainly been demonstrated at high intakes, and several studies have found an average intake of fructose to cause no health problems [12, 110, 111, 160]. Although the average daily intake of fructose is 50–60 g/day, some of the population will consume larger amounts [161, 162], so the negative health effects of fructose may be relevant at least for a proportion of the population. Many of the current studies are poorly suited to determine health risks of fructose because (a) the fructose intakes are unrealistically high, (b) fructose is given in isolation and not mixed with other carbohydrates as in practice, and/or (c) the studies are conducted on animals. Differences between human and animal physiology limit their applicability to humans. There is a need for more human studies under conditions more similar to the way fructose is normally consumed. Such studies, particularly related to DNL, will be necessary to understand the effects of a normal fructose intake. The effects of fructose on triglyceride and cholesterol levels in the blood, fat accumulation in liver, and insulin signaling all seem to be linked to the extent to which fructose enters DNL. Surprisingly, there have been no satisfactory studies to assess the proportion of fructose that enters DNL at different levels of intake. Such studies should be carried out and should include intake of both pure fructose and fructose together with glucose.

It is also important to note that, despite the metabolic difference between glucose and fructose, glucose consumption far exceeds fructose consumption in the human diet [12]. This quantitative aspect must be considered when comparing the health effects of glucose and fructose.

## 9. Conclusion

Although there is a paucity of published literature regarding physiological effects of fructose in humans, current literature does not indicate that a normal consumption of fructose (approximately 50–60 g/day) increases the risk of atherosclerosis, type 2 diabetes, or obesity more than consumption of other sugars. However, a high intake of fructose, particularly if combined with a high energy intake in the form of glucose/starch, may have negative health effects via DNL. More studies are clearly needed, particularly studies under more realistic consumption levels of fructose.
